# A scoping review of bicycling interventions’ impacts on psychological, social, affective, and cognitive well-being

**DOI:** 10.3389/fspor.2026.1807791

**Published:** 2026-05-19

**Authors:** Lauren Schuck, Brandie Reisman, Esther Walker, Seth Wiafe, Sean M. Wilson, Cian L. Brown

**Affiliations:** 1 Outride, Morgan Hill, CA, United States; 2 Department of Educational Psychology, The University of Oklahoma, Norman, OK, United States; 3 School of Public Health, Loma Linda University, Loma Linda, CA, United States; 4 School of Medicine, Loma Linda University, Loma Linda, CA, United States

**Keywords:** bicycling, cognitive function, cycling, intervention, mental health, mountain biking, physical activity, psychological well-being

## Abstract

**Introduction:**

Bicycling, inclusive of cycling on trail or road, and stationary forms, is recognized for its substantial physical benefits and has gained attention for its broader impact on dimensions of well-being. Evidence demonstrates that bicycling improves cardiovascular fitness, body composition, and metabolic health while serving as an effective strategy for exercise promotion and disease prevention. Beyond physical outcomes, growing evidence supports bicycling's impact on psychological and cognitive domains of well-being from large associative studies, active travel, and bicycling programs.

**Methods:**

This scoping review synthesizes research examining the impact of bicycling interventions on psychological, social, affective, and cognitive domains of well-being. Database searches of EBSCOhost, ProQuest, PsycINFO, PsycArticles, and PubMed identified relevant studies. Of the 1,653 studies identified, 87 studies met inclusion criteria.

**Results:**

A majority of studies implemented acute, indoor bicycling interventions assessing cognitive outcomes. Synthesized results indicate positive impacts of bicycling on well-being, including improved mood, reduced depressive symptoms, increased social connection, and enhanced cognitive functioning, especially among interventions occurring outdoors and over multiple sessions. Affective and cognitive outcomes varied by intervention, context, and population.

**Discussion:**

This scoping review reinforces bicycling as a multidimensional modality for promoting well-being by synthesizing findings from 87 intervention studies across 19 countries. Notable improvements across well-being domains emerged, particularly in multi-session, outdoor interventions. Findings underscore the necessity for translational and inclusive community-based research beyond indoor environments, and exploration of how unique bicycling features (i.e., outdoor exposure, social opportunity, and meaningful routine) promote holistic well-being across the lifespan.

## 1 Introduction

Bicycling, whether on mountain or trail, road, or stationary bikes, and whether for transportation, recreation, or competition, has been widely studied for its contributions to physical, emotional, and psychological well-being (1, 2). As interest grows in the relationship between movement and mental health, bicycling has emerged as a practical, accessible, adaptable, and sustainable activity that supports holistic wellness across diverse populations and settings (3).

Beyond its well-documented physical health benefits (4), bicycling has been increasingly recognized for its positive effects on mental and emotional well-being (2, 5). Physical activity, including bicycling, has been associated with reduced symptoms of depression and anxiety, improved mood, and enhanced emotional regulation (3, 6). Neurobiological mechanisms, such as the release of endorphins and other neurotransmitters, may underlie some of these effects (7). Additionally, bicycling often facilitates social interaction and community engagement, contributing to greater social cohesion and a sense of belonging (8, 9).

Much of the existing literature relies on population-level survey data to examine associations between bicycling and mental well-being (10, 11). For example, Friel et al. (12) conducted a prospective longitudinal study and found that commuting via bicycle was associated with a lower risk of all-cause mortality, as well as a lower likelihood of having a prescription for mental health problems compared to non-bicyclist commuters. These studies offer important evidence on the role bicycling may play in supporting well-being, but they are limited in their ability to establish causal relationships. In contrast, intervention-based studies, which involve structured programs or activities designed to assess the direct impact of bicycling activities, can provide stronger evidence of the intentional effects of bicycling on specific health outcomes. Yet, to date, these intervention studies remain fragmented and under-synthesized. This scoping review aims to address this gap by systematically mapping the existing literature on bicycling-related interventions and their impacts on psychological, social, affective, and cognitive well-being. By focusing specifically on interventions, we seek to better understand how bicycling can be intentionally leveraged to promote these domains of well-being across the lifespan.

Several frameworks describe the interaction between physical activity and cognitive, psychological, and behavioral outcomes. Self-determination theory suggests physical activity enhances well-being by satisfying core psychological needs for autonomy, competence, and relatedness (13). Whereas a biopsychosocial model suggests that psychological benefits, such as improvements in mood, are promoted by physiological changes, such as the release of endorphins that occur during and after physical activity (14). Together, these theories highlight the complex interplay between physical activity and various domains of well-being. In practice, bicycling programs may engage multiple domains of well-being from individual to community levels (15). However, despite these frameworks, how bicycling interventions are intentionally designed to leverage these mechanisms, particularly for non-physical outcomes, has yet to be synthesized.

Given the range in how bicycling interventions are researched, designed, implemented, and evaluated across different populations and outcome domains, a scoping review is warranted to map the available evidence, identify key themes, and highlight gaps in the literature. For the purposes of this review, we adopt a broad definition of “bicycling interventions” as the purposeful or structured use of a bipedal vehicle (e.g., road, mountain, gravel, and other bicycles used outdoors or indoor stationary bicycles) to facilitate changes across four domains of well-being (psychological, affective, social, and cognitive, see Table 1). Under this definition, the included interventions span a wide range of aims, intensities, time scales, and settings. For example, indoor, laboratory-based acute cycling studies often prioritize internal validity to isolate specific effects and mechanistic insight. On the other hand, multi-session outdoor programs often prioritize ecological context and real-world implementation. Including these dimensions (i.e., indoor vs. outdoor; acute vs. multi-session) allows this review to map the full range of pathways through which bicycling may influence well-being. All age groups, clinical and non-clinical populations, and international studies published in English were included to capture global trends and translational work.

**Table 1:** Descriptions of the domains of well-being. The table describes the domains of well-being and provides examples of related subareas and measures used to assess each domain.

Well-Being Domain	Description	Examples of Subareas	Example Measures from the Literature
Psychological Well-Being	The broader state of mental and emotional health, including self-esteem, purpose, and self-efficacy.	Self-esteem, self-efficacy	36-Item Short Form Health Survey (22), General Self-Efficacy Scale (29), World Health Quality Assessment Instrument-100 (31)
Social Well-Being	Social well-being refers to how individuals perceive the quality of their relationships and their integration within social systems, including family, peers, and communities. Strong social wellness is associated with stronger support networks and a reduced risk of mental health challenges such as loneliness and depression.	Feelings of social support, feelings of isolation, sense of belonging, community engagement.	Trier Social Stress Test for Groups (34), Social Interaction Anxiety Scale (36), Lubben Social Networking Scale (40)
Affective Well-Being	Affective well-being concerns the balance of positive and negative emotions and mood. It is a core component of subjective well-being and closely tied to emotional regulation capacities.	Mood, depressive and anxiety symptoms, emotional regulation.	Positive and Negative Affect Schedule (61), Beck Depression Inventory (47), State and Trait Anxiety Inventory (53)
Cognitive Well-Being	Cognitive health refers to the ability to think, learn, and remember, encompassing mental functions such as attention, memory, processing speed, executive function, and problem-solving. It is an important component of overall brain health. Maintaining strong cognitive health is especially important across the lifespan to prevent cognitive decline and support adaptive functioning.	Attention, memory, and executive functioning; cognitive flexibility and processing efficiency; risk of cognitive impairment.	Wisconsin Card Sorting Test, Stroop Task, N-Back Task, Tower of London, Trail Making Test, Digit Span, Auditory Oddball, Task (P300) EEG, fMRI, fNIRS

This comprehensive approach aligns with the goals of this scoping review to synthesize what is known about bicycling interventions, highlight what dimensions may shape different outcomes, and identify gaps to inform future research and practice. Specifically, this review addresses how bicycling interventions differ across indoor and outdoor settings, how acute, short-term, and long-term interventions assess different domains of well-being, and which populations are represented across the literature.

## 2 Methods

### 2.1 Data sources and search strategy

This scoping review was conducted by integrating the Joanna Briggs Institute (JBI) methodology (16) with the Preferred Reporting Items for Systematic Reviews and Meta-Analyses extension for Scoping Reviews (PRISMA-ScR) (17). A comprehensive search strategy was utilized to identify literature examining the relationship between bicycling (including mountain biking, road biking, and stationary biking) and four domains of well-being including psychological, social, affective, and cognitive well-being. A database search of EBSCOhost (Academic Search Complete, Academic Search Elite, Academic Search Premier, CINAHL Complete, ERIC, MEDLINE, Psychology and Behavioral Sciences Collection), ProQuest, PsycINFO, PsycArticles, and PubMed was completed using key search terms. The following search string was implemented:

“Mountain Biking” OR “Road Biking” OR “Road Cycling” OR “Bicycling” BY TITLE OR ABSTRACT AND “Mental Health” OR “Mental Wellbeing” OR “Health” OR “Wellbeing” OR “Physical Health” OR “Physical Wellbeing” OR “Psychological Wellbeing” OR “Psychological Health” OR “Cognitive” OR “Neurological” OR “Physical” OR “Social Emotional Health” OR “EEG” BY ABSTRACT

Medical Subject Headings (MeSH) terms “Bicycling/physiology” OR “Bicycling/psychology” were also incorporated into the EBSCOhost and PubMed searches; however, such options were not available through PsycINFO or ProQuest. To ensure relevancy and recency, the publication date range was limited to a 20-year period ranging from September 27, 2004, to September 27, 2024, based on the date of the initial search. The final search was conducted on February 20, 2025.

### 2.2 Study inclusion criteria

The research question was developed using The Population, Concept, and Context (PCC) framework (16). For this Scoping Review, the population consisted of individuals of all ages and included clinical populations; the concept was any bicycling intervention that assessed psychological, social, affective, and/or cognitive well-being outcomes; the context included all geographic locations and participant demographics so long as the bicycling intervention primarily assessed one of the above domains of well-being. Studies that were not accessible in English were excluded. Additional details regarding study design are reported in Table 2.

**Table 2:** Characteristics of included studies: The table summarizes characteristics of the 87 studies included in this scoping review reporting data for the author and year, country, number of participants (n), intervention design, Well-Being Domain(s) assessed, selection of well-being measures, highlight of results, type of bicycle used, and whether the intervention primarily occurred indoor or outdoor.

Author, Year	Country	n	Intervention	Well-Being Domain(s)	Selection of Measures	Results	Bike, Location
Aguirre-Loaiza et al. (63)	Colombia	19	2 Groups (Physical Exercise group of 1 session of 20 min cycling, Control: rest)	Cognitive	Stroop Task	Reduced RTs in Physical Exercise Group	Stationary, Indoor
Akaiwa et al. (82)	Japan	25	4 Conditions (Only oddball task; Oddball task while pedaling at optimal cadence; Oddball task while pedaling faster than optimal; Oddball task while pedaling slower than optimal)	Cognitive	Oddball Task, EEG (P3)	P300 amplitude at Fz decreased under fast and slow conditions; P300 amplitudes at Pz under fast and slow conditions were significantly lower than those under the optimal condition	Stationary, Indoor
Anderson et al. (44)	Australia	20	12-week trial of bicycle commuting	Affective; Social	Qualitative Interviews	E-cycling participants reported improved mental and physical well-being	E-Bike, Outdoor
Anderson-Hanley et al. (120)	United States	10	2 Groups (stationary bicycle or a cybercycle); 2–5×/week 45 min per session for 3 months	Cognitive	Trail Making Test (TMT), Stroop Test, Digit Span, Purdue Pegboard (PP)	No significant differences in executive function; exercisers with Diabetes exhibited significant gains in executive function	Stationary or Cybercycle, Indoor
Ando et al. (64)	Japan	12	3 Sessions (1 calibration, 2 experimental); 2 Conditions (Cycle at 40%, 60%, and 80% of VO2, Rest fixed at 20W) cycling for about 20 min	Cognitive	Flanker Task, NIRS System	Premotor time during exercise at 60% Vo2 significantly shorter relative to that at rest, but no differences at 80%; cerebral oxygenation substantially decreased during exercise at 80% peak Vo2	Stationary, Indoor
Armstrong et al. (45)	Australia	29	3 Conditions (FES-cycling, adapted cycling and goal-directed training); 3 one-hour training sessions/week for 8 weeks	Affective; Social	Qualitative Interviews	Themes: engagement facilitated by the therapist's connection, cycling is fun and participant driven goal setting, while getting there and time off school were identified as challenges. Participants positively linked improved physical function to greater independence.	Stationary, Indoor
Avila-Gandia et al. (121)	Spain	25	2 constant intensity cycling tasks with different cognitive loads	Affective; Cognitive	n-back, oddball, self-assessment manikin (SAM)	Higher executive load elicited less positive exercise-triggered valence	Stationary, Indoor
Brookman et al. (28)	Australia	32	26-day virtual cycling competition	Affective; Cognitive; Psychological; Social	Geriatric Depression Scale (GDS), The Geriatric Anxiety Inventory (GAI), Montreal Cognitive Assessment (MoCA); The Cornell Scale of Depression in Dementia (CSDD), The General Self Efficacy Scale (GES), Lubben Social Networking Scale (LSNS)	Significant decrease in depression, increase in self-efficacy, self-reported improvements in well-being	Stationary, Indoor
Brown et al. (5)	United States	41	2 Groups (Adventure Therapy Mountain Bike, Mountain Bike); 14 Sessions: 9 Weeks: 2×/week 90 min	Affective; Psychological; Social	Qualitative Interviews	Mountain bike program increased focus, competency, physical and mental wellbeing, and connection to the environment	MTB, Outdoor
Brown et al. (104)	United States	41	2 Groups (Adventure Therapy Mountain Bike, Mountain Bike); 14 Sessions: 9 Weeks: 2×/week 90 min	Psychological	Resiliency Scales for Children and Adolescents (RSCA)	Participants in the Adventure Therapy only group experienced an increase in resiliency, while those in the MTB only group experienced a decrease.	MTB, Outdoor
Buchholtz & Burgess (122)	South Africa	35	1 session; Cycle at 3 different intensities for 10 min each (30 min total)	Cognitive	Eye Gym Reaction Time Test	There were no significant differences in SRT or CRT between sexes and no significant differences in SRT at different intensities of cycling for all participants; significant difference between CRT at different intensities	Stationary, Indoor
Bullock et al. (65)	United States	12	3 conditions: rest, low-intensity exercise, and high-intensity exercise of 50 min each	Cognitive	Oddball Task, EEG (P3)	RT significantly decreased as a function of exercise intensity with fastest in high intensity condition; target detection accuracy was not modulated by exercise	Stationary, Indoor
Bullock et al. (123)	United States	18	2 Sessions (Baseline, Cycling: Low Intensity and High Intensity for 40 min each)	Cognitive	EEG Cortical Activity, Orientation Test	Feature-selective tuning profiles were highest during low-intensity exercise	Stationary, Indoor
Davranche et al. (66)	UK	14	2 periods of 20-min cycling at 50% maximal aerobic power	Cognitive	Flanker Test	Faster reaction time	Stationary, Indoor
Dementyev et al. (116)	United States	1,148	6–8-week middle school intervention; ∼20 min cycling 3 days/week	Affective; Psychological	WHO-5 Well-Being, The Pediatric Symptom Checklist-17 (PSC-17-Y)	General increase in psychosocial well-being	MTB, Outdoor
Dishman et al. (60)	United States	36	3 Conditions (moderate intensity aerobic exercise, low intensity aerobic exercise, or no-treatment control); 3 x/week for 30 min over 6 weeks	Affective; Cognitive	Profile of Mood States-Short Form (POMS-SF), EEG Cortical Activity	EEG activity was not different between groups or across time over anterior sites	Stationary, Indoor
Dodwell et al. (88)	Germany	24	3 Conditions (Perform a task at rest, during moderate-intensity exercise, and during vigorous-intensity exercise) lasting ∼25 min each	Cognitive	EEG (N2), Additional-Singleton Visual Search Task	Faster reaction times during vigorous exercise	Stationary, Indoor
Duchesne et al. (50)	Canada	39	2 Groups (Healthy Control, Parkinson's Disease); 36 Sessions: High-intensity, stationary recumbent bike-training program, 3×/week for 12 weeks) for 20–40 min	Affective; Cognitive	Stroop Test, Serial Reaction Time Task (SRTT), Beck Depression Inventory (BDI), Beck Anxiety Inventory (BAI), Montreal Cognitive Assessment (MoCA)	Aerobic Exercise Training improved inhibition and motor learning skill in both groups	Stationary, Indoor
Dutke et al. (74)	Germany	60	4 groups: Physical load (medium vs. high) and Cognitive load (lower vs. higher); Cycle for 18 min in each session	Cognitive	Word Comparison Test, Interval Production	No significant differences for accuracy or response times	Stationary, Indoor
Enders et al. (92)	Canada	10	3 Sessions (1 maximum aerobic power test, 2 time-to-exhaustion trial at 85% maximum power output, separated by 48 h)	Cognitive	EEG Cortical Activity	Left frontal cortex showed an increase in alpha, beta, and gamma bands; SMA and left parietal cortex significant increase in the alpha and beta bands; right parietal cortex only for the alpha band	Stationary, Indoor
Fiorelli et al. (124)	Brazil	12	3 Conditions (Control session; HIIT: 25-minutes of high-intensity cycling intervals, and MICT: 0-minute moderate intensity cycling)	Cognitive	WAIS III, Symbol Search, Digit Span, Trail Making Test (TMT)	MICTimproved immediate auditory memory; HIITimproved immediate auditory memory, attention, and sustained attention	Stationary, Indoor
Focht et al. (25)	United States	33	2 Groups (Young adult, Older adult); 20-min bout of stationary cycling at 65% of VO2peak	Affective; Psychological	Feeling Scale and Felt-Arousal Scale, Exercise-Induced Feelings Inventory, 4-Item Self Efficacy Questionnaire	Both groups reported reduced pleasant feeling states and self-efficacy and increased physical exhaustion	Stationary, Indoor
Fontes et al. (101)	South Africa	7	Cycling in MRI Machine: 6 trials of 2 min with 16-s intervals between trials	Cognitive	fMRI	Cerebellar vermis and precentral and postcentral gyrus activated when cycling; Posterior cingulate gyrus and precuneus activated during challenging blocks	Stationary, Indoor
Geard et al. (38)	Australia	26	2 Groups (Cycling training: 3 sessions per week of 45–60 min cycling vs. Recreation: Bicycle as usual); 12 weeks	Cognitive; Psychological; Social	The Veterans RAND 36-Item Health Survey (VR-36), UCLA 3-item Loneliness Scale, MOS Cog-R	Training Group had significantly higher social functioning	Stationary, Indoor
Grego et al. (125)	France	12	2 Conditions (Control, Cycling); 2 Session: baseline, 3 h cycling exercise cycling at 66% VO2 max	Cognitive	Auditory Oddball, EEG (P3), Cortisol	Temporary increase in P300 between 1st and 2nd hour, Increased cortisol	Stationary, Indoor
Grego et al. (67)	France	8	2 Groups (Control and Cycling: 3 h cycling task at a∼ 60% of VO2max); 2 Sessions (fluid ingestion, no fluid ingestion)	Cognitive	Critical Flicker Fusion test (CFF), Map recognition	Decrease in CFF performance and increased errors after 120 min of exercise when compared with the first 20 min; Faster RTs between 20 and 120 min	Stationary, Indoor
Harper et al. (49)	United States	35	2 Groups (Cycling, Control); 3 Sessions: Cycle for 40 min, 1 week	Affective; Cognitive	Beck Depression Inventory (BDI-II), Montreal Cognitive Assessment (MoCA); Val66 polymorphism	Significant main effect of time for emotion recognition	Stationary, Indoor
Harwood et al. (76)	United States	10	5 sessions (1 calibration, 4 bicycling for 25 min at different intensities; 5 weeks	Cognitive	Oddball paradigm. Montreal Cognitive Assessment (MoCA), EEG (P3)	Bigger P300 associated with post-exercise recumbent bike cycling	Stationary, Indoor
Hazamy et al. (68)	United States	60	2 Sessions (Dual Task: Cognitive task while pedaling at self-paced speed; Single Task: No cycling)	Cognitive	Cognitive Test Battery	Dual task: faster RTs in visual tasks across cognitive domains, and improved verbal recall; Persons with Parkinson's Disease exhibited impairments compared to healthy participants in select tasks	Stationary, Indoor
Holzapfel et al. (126)	United States	34	3 Groups (Assisted Cycling Therapy, voluntary cycling, and no cycling); 24 Sessions: 8 weeks, 30 min sessions 3×/week	Cognitive	Tower of London (TOL), Peabody Picture Vocabulary Test 4th ed. (PPVT-IV), Purdue Pegboard (PP)	Assisted Cycling Therapy improved dexterity, temporal and spatial processing, and cognitive planning	Stationary, Indoor
Holzschneider et al. (102)	Germany	106	2 Groups (aerobic endurance training (cycling) or a non-endurance training (stretching and coordination)); 60 min cycling 2x/week for 6 months;	Cognitive	fMRI, Virtual Maze Task	Behavioral data did not differ between the cycling and the stretching group	Stationary, Indoor
H**ö**tting et al. (127)	Germany	68	3 Groups (Cycling Training: 45 min each session, Stretch/Coordination, Sedentary Control); Twice a week for 6 months.	Cognitive	Auditory Verbal Learning Test, Zahlenverbindungstest (Similar to Trail Making Test), “d2” Letter Cancellation Test, Stroop Task, Leistungsprufsystem Intelligence Test	Significant improvements in memory were observed in both the cycling and the stretching/ coordination group as compared with the sedentary control group.	Stationary, Indoor
Hüttermann & Memmert (128)	Germany	17	2 Groups (Expert athletes, non-athletes) cycle at 50%, 60%, and 70% of the maximum heart rate for 10 min each	Cognitive	Visual Field Test, Attentional Breadth Task	Expert athletes outperformed non-athletes in the attentional breadth task during 50% workload; participants attained highest success rates at 60%	Stationary, Indoor
Joubert et al. (129)	United States	21	2 Groups: traditional sit (SIT) and stationary cycle (CYC) groups; CYC pedaled 10 min during a 50-min lecture, 3x/week for 12 weeks	Cognitive	Grade Point Average (GPA), Academic Performance	No significant differences between CYC and SIT on in-class test scores or overall course grades	Stationary, Indoor
Kilpatrick et al. (130)	United States	24	5 sessions; 4 conditions of 20 min at different cycling intensities separated by at least 24 h	Affective	11-point Feeling Scale (FS), 7-point Exercise Enjoyment Scale (EES)	Affective and enjoyment responses were significantly less positive for the heavy continuous trials	Stationary, Indoor
Kojima et al. (99)	Japan	12	2 Conditions (Rested wakefulness, 24-h Sleep Deprivation); Pre and Post cognitive assessment; 20-min cycling at 60% VO2peak	Cognitive	Stroop Test, NIRS	Oxygenation to the DLPFC increased at 12 min after exercise onset; moderate-intensity exercise reverses Sleep Deprivation-induced cognitive decline	Stationary, Indoor
Leyland et al. (19)	UK	100	3 Groups (Non-Cycling Controls, Pedal Cyclists, E-Biker Cyclists); Cycle 3x/week for at least 30 min over 8 weeks	Affective; Cognitive; Psychological	Stroop Task, Letter Updating Task, Psychological Well-being Qx (PWB); Positive and Negative Affect Schedule (PANAS); Consortium to Establish a Registry for Alzheimers Disease (CERAD) immediate and delayed recall. MMSE; Satisfaction in Life (SL), Health Survey Short Form (SF-36)	Both cycling groups improved in accuracy compared to non-cycling control participants. E-bike participants also improved in processing speed and in their mental health scores (SF-36) compared to non-cycling controls	E-Bike, Outdoor
Li et al. (35)	China	80	2 dyad groups (Experimental: 30-min bicycling; Control: 30 min single person sitting); 30-minutes over 1 session	Affective; Cognitive; Social	fNIRS, Prisoner's Dilemma, Social Interaction Anxiety Scale (SIAS), Beck Depression Index (BDI)	Cycling dyads had significantly higher cooperation rate and efficiency after intervention.	Stationary, Indoor
Lindheimer et al. (56)	United States	60	4 Groups (Active cycling/informed on benefits; Active cycling/not informed; Passive cycling/informed, Passive cycling/not informed); Active cycling: 30 min at 35% V02 Max; Passive Cycling: 30 min	Affective; Cognitive	State Trait Anxiety Inventory (STAI); Profile of Mood States (POMS-BF): Visual n-back Task	Mood and cognitive performance were not improved by active or passive cycling	Stationary, Indoor
Ludyga et al. (93)	Germany	26	1 session; Cycle for 30 min	Cognitive	EEG Cortical Activity	No observed sex-specific differences of cortical activity during exercise at constant workload.	Stationary, Indoor
Ludyga et al. (94)	Germany	29	2 Groups (High V02, Low V02); 2 Sessions (1: Graded cycling exercise over 40 min, 2: EEG recording at rest and cycling for 30 min)	Cognitive	EEG Cortical Activity	Alpha/beta ratio was increased in HIGH Vo2 compared to LOW V02 in resting state and exercise condition	Stationary, Indoor
Ludyga et al. (97)	Germany	22	2 Groups (low and high cycling cadence training); 60 min of cycling per week over 4 weeks	Cognitive	EEG Cortical Activity	Greater increase of frontal alpha/beta ratio was confirmed in high cadence group	Stationary, Indoor
Micklewright et al. (131)	United States	20	2 laboratory ramped cycling tests beginning at 50 W and increasing by 0.25 W/s until volitional exhaustion	Cognitive	Recognition Memory Task	Recognition memory performance had strong negative correlations with perceived exertion	Stationary, Indoor
Miki et al. (132)	Japan	78	2 Groups (Intervention: speed-feedback therapy with a bicycle ergometer 1×/ week for 4 weeks, Control: life as normal); Cycle for 5 min	Cognitive	Frontal Assessment Battery; Functional Assessment of Cancer Therapy General (FACT-G)	Frontal Assessment Battery for the intervention group was higher than control group at week 4; No differences in FACT-G	Stationary, Indoor
Miyamoto et al. (133)	Japan	13	4 sessions of 30 min cycling (Control, Cognitive and Physical Exercise, Cognitive Exercise, Physical Exercise)	Cognitive	Stroop Test, Digit Span, Wisconsin Card Sorting Task (WCST), BDNF	CCPE had no additional or synergistic effect on peripheral BDNF levels compared to PE alone.	Stationary, Indoor
Moore et al. (75)	United States	30	2 Groups (Exercise Group with 60-min bout of cycle ergometry at 90% ventilatory threshold, Rest Group for 60 min)	Affective; Cognitive	Mental and Physical State and Trait Energy and Fatigue Scale, Visual-discrimination test, Vigilance test	Exercise Group had slower perceptual-discrimination tasks and response times	Stationary, Indoor
Mullane et al. (134)	United States	9	4 conditions (SIT, STAND, WALK and CYCLE) across a 6 h. period with a 7 day washout period between conditions.	Cognitive	One Back Test, Set Shifting Test, Detection Test	CYCLE condition had most pronounced Treatment effect	Stationary, Indoor
Neumeier et al. (20)	Austria	74	2 Groups (Active Commuting, Control: No change); 12 months	Psychological	Health Survey Short Form (SF-36)	Active commuting group reported improved mental health and quality of life	2 Wheel, Outdoor
Olson et al. (69)	United States	27	3 Sessions: 1: Exercising on a cycle ergometer at 40%, 2: Cycle 60% VO2 peak, 3: No-exercise seated control	Cognitive	Flanker task, EEG (N2, P3)	Impaired accuracy during both exercise conditions; Faster reaction times during moderate-intensity exercise.	Stationary, Indoor
Olson et al. (51)	United States	27	2 Conditions (low intensity cycling for 20 min, seated control); 2 Sessions	Affective; Cognitive	EEG (P3), Visual oddball, State Trait Anxiety Inventory (STAI); Perceived Stress Scale (PSS), Beck Depression Inventory (BDI)	Significant reduction in P300 amplitude was observed during the 20 min exercise period compared to the control condition	Stationary, Indoor
Page & Nilsson (41)	UK	31	2 Groups (Active Commuting, Travel as Usual); 3–8-week E-Bike Loan	Affective; Psychological; Social	Organizational Citizenship Behavior (OCB), Counterproductive Workplace Behavior (CWB), Flourishing scale, Short General Health Questionnaire (GHQ12), Weekly diary	Active commuting reported more positive affect, better physical health and more productive organizational behavior outcomes; more frequent active commute was positively associated with more productive organizational behavior and stronger overall positive employee well-being	E-Bike, Outdoor
Palmer et al. (135)	United States	300	3 groups (Mountain Biking Unit Intervention, Standard PE, No PE); 4 weeks	Psychological	Behavioral Regulation in Exercise Questionnaire (BREQ), Measure of perceived self-efficacy for overcoming barriers, Physical Self Perception Profile	No main effects of the PE intervention	MTB, Outdoor
Pasekova et al. (95)	Russia	30	1 session; Increasing Cycling Load test until submaximal heart rate	Cognitive	EEG Cortical Activity	Gradual Alpha power increase was detected in both hemispheres with every load stage	Stationary, Indoor
Perciavalle (136)	Italy	30	Multistage incremental cycling test to exhaustion	Cognitive	Self-Ordered Pointing Task (SOPT)	Acute exhaustive exercise increases blood lactate and is associated with a significant worsening of WM.	Stationary, Indoor
Pesce et al. (70)	Italy	16	2 Groups (Physically active vs. inactive older adults) complete acute cycling task with attentional task; 2 acute sessions	Cognitive	Go/No-go task	Physically active individuals had faster RTs	Stationary, Indoor
Pickering et al. (46)	UK	35	2 Groups (Cycling, Control Waitlist) for 6 weeks	Affective; Social	Qualitative Interviews	Interview themes: Learning a new skill; impact on wider family and friends; opportunity for social participation; future aspirations	2 wheel; Adaptive, Outdoor
Pontifex & Hillman (79)	United States	41	2 Conditions (Upright cycling at 60% of max HR; Rest); Cycle ∼10 min	Cognitive	Flanker Test, EEG (N1, N2, P2, P3)	Exercise: Reduced accuracy for incongruent trials; Decreased N1 at parietal sites and globally for N2	Stationary, Indoor
Proost et al. (137)	Belgium	16	3 Sessions: 1 calibration, 2 experimental of low-intensity bike task for 9 min; 2 conditions: cycle after mentally fatiguing task or rest	Cognitive	Flanker Task, Stroop Test, EEG Cortical Activity	Higher mental workload in mentally fatiguing conditions compared to rest; Significant differences in beta frequency during biking	Stationary, Indoor
Qi et al. (138)	China	79	3 groups: control group (CG), dancing group (DG) and bicycling group (BG) of 30 min cycling; 12 weeks	Cognitive	EEG Cortical Activity	Brain flexibility across all recording sites and four brain regions in both the DG and BG groups were significantly enhanced.	Stationary, Indoor
Quaney et al. (77)	United States	38	2 Groups (Cycle Group: Progressive resistive stationary bicycle training at 70% max heart rate, Stretching Exercise Group: stretches at home); 24 Sessions: 3×/week, 45-min sessions, 8 weeks	Cognitive	Wisconsin Card Sorting Task (WCST), Stroop Test; Trail Making Test (TMT), Serial Reaction Time Test (SRTT); Predictive Grip Force Modulation (PGFM), Fugl-Meyer Sensorimotor Test	AEX significantly improved information processing speed, predictive force accuracy	Stationary, Indoor
Ridgel et al. (73)	United States	19	4 sessions 1×/ week for 4 weeks; 3 30 min passive cycling sessions on motorized bicycle	Cognitive	Trail Making Test (TMT)	Significant improvement on the TMT-B after passive leg cycling; Decreased RTs from pre to post	Stationary, Indoor
Ringenbach (26)	United States	9	3 sessions; 3 Conditions (voluntary cycling (VC); assisted cycling (AC): no cycling (NC)); Each intervention separated by 1 week; 30 min exercise	Cognitive; Psychological	Purdue Pegboard (PP), Physical Activity and Self-efficacy, Tower of London	RTs decreased and TOL improved for Assisted Cycling	Stationary, Indoor
Ringenbach (139)	United States	44	3 sessions per week for 8 weeks; 3 Groups (Assisted Cycling, Voluntary Cycling, Control); 35 min cycling per session	Cognitive	Wisconsin Card Sorting Task (WCST), Visual Choice Reaction Time (VCRT); NEPSY Knock-Tap Task, Language Fluency	The Assisted Cycling group showed significantly improved RTs	Stationary, Indoor
Ryu et al. (30)	Republic of Korea	60	2 (Groups Outdoor Cycling, or Occupational Therapy); 16 weeks with one 90-min group session per week, 40 min of cycling	Affective; Cognitive; Psychological	Brief psychiatric Rating Scale (BPRS), Beck Depression Index (BDI), State Trait Anxiety Inventory (STAI), Rosenberg Self-Esteem Scale (RSE), Wisconsin Card Sorting Test (WCST), The World Health Organization Quality of Life Scale (WHOQOL-100), The Global Assessment of Functioning Scale (GAF)	Outdoor Cycling Group: Improved psychotic symptoms, depression, state and trait anxiety, and global functions	2 wheel, Outdoor
Samendinger et al. (24)	United States	82	12 cycling sessions over 4 weeks; Cycle at 75% Max Heart Rate 50 min or less	Psychological	Exercise Persistence, Self Efficacy Questionnaire	Previous days’ residualized performance was a significant predictor of performance, as was same-day residualized self-efficacy; Residualized self-efficacy became a stronger predictor over time	Stationary, Indoor
Sandroff et al. (71)	United States	24	5 sessions (1 calibration, 4 experimental); 4 Conditions (Cycling: Incremental maximal exercise cycling test for 20 min; Walking; Guided Yoga; Rest); 5 weeks	Cognitive	Flanker Test, Symbol Digit Modalities Test (SDMT), Neurological Exam	General pre-to-post improvements in reaction time, but not accuracy for all 3 exercise modalities but not rest	Stationary, Indoor
Scanlon et al. (86)	Canada	12	2 conditions: inside cycle, outside cycle for 24 cycling min each	Cognitive	Auditory Oddball, EEG (P, N1, P2)	Significantly reduced P300 difference in the outdoor cycling; Significantly larger average amplitude of the N1 for outside	2 wheel, Outdoor
Scanlon et al. (78)	Canada	10	2 Conditions (Quiet Park, Busy Roadway); Cycle for 24 min	Cognitive	Auditory Oddball, EEG (N1, P3)	Increased N1 near roadway; no significant difference in response accuracy; significant increase in beta oscillations in park condition	2 wheel, Outdoor
Schmidt-Kassow et al. (90)	Germany	12	2 Groups (Exercise: 30 min, No Exercise); 3 weeks	Cognitive	EEG (N4), Vocabulary Test	Larger N400 effect and better performance in vocabulary tests when physically active during the encoding phase. Spinning group outperformed sitting group	Stationary, Indoor
Schmidt-Kassow et al. (140)	Germany	105	2 Groups (No exercise, Exercise: 30 min bicycle); 2 sessions within 48 h	Cognitive	BDNF, Vocabulary Learning	Light- to moderate-intensity physical activity during encoding improved vocabulary learning	Stationary, Indoor
Schmidt-Kassow et al. (84)	Germany	17	1 session; 4 experimental blocks (pedaling periodic; pedaling aperiodic; sedentary periodic; sedentary aperiodic)	Cognitive	EEG (P3)	Simultaneous pedaling increased predictability of P300	Stationary, Indoor
Shima et al. (59)	Japan	14	3 Conditions over 3 Sessions: non-exercise (Rest), standard solitary cycling exercise (Ex), and AR-based multi-person cycling exercise (Ex + AR) for 10 min	Affective; Cognitive	Profile of Mood States 2nd Edition (POMS 2), Oxytocin	Ex + AR significantly suppressed the depression-ejection score and significantly increased salivary oxytocin levels	Stationary, Indoor
Storzer et al. (141)	Germany	14	2 Conditions (Cycle, Walk); 20-minute sessions	Cognitive	EEG Cortical Activity	Bicycling associated with decrease in high beta band (23–35 Hz) during movement initiation and execution	Stationary, Indoor
Storzer et al. (52)	Germany	13	3 Conditions (stationary bicycle simulator, over-ground walking, baseline control); 7 total minutes of movement	Affective; Cognitive	Mattis Dementia Rating Scale (DRS), the Frontal Assessment Battery, and the Beck Depression Inventory (BDI)	Both bicycling and walking led to a suppression of subthalamic beta power (13–35 Hz), and this suppression was stronger for bicycling.	Stationary, Indoor
Szabo et al. (62)	Hungary	18	2 conditions: No instructor, instructor-led workout; 2 sessions of 35 min cycle	Affective	Positive and Negative Affect Schedule (PANAS), Subjective Enjoyment Scale	Positive affect increased while negative affect decreased after both workouts; participants enjoyed more the instructor-led exercise session	Stationary, Indoor
Tempest & Reiss (98)	United States	13	4 Sessions: 1 calibration session, 3 cycling sessions at different intensities for 16 min	Cognitive	0 and 2 back Visuospatial n-back test, fNIRS	Consistent cortical activation in the contralateral primary motor cortex while cycling at a low and high intensity	Stationary, Indoor
Theurel et al. (142)	France	10	2 Conditions: E-Bike vs. Classic Bike; 3 sessions (1 calibration, 2 cycling in each condition for 30 min)	Cognitive	Mail Sorting Test	Mail sorting time significantly longer after exercise with classic bikes compared to cycling with E-Bike or rest	Traditional and E-Bike, Indoor
Tollár et al. (21)	Netherlands	83	3 Groups (High-intensity agility exergaming, Stationary cycling, Control: No exercise); 30 sessions: 5×/week for 5 weeks cycling at 80% of max heart rate for 1 h	Affective; Psychological	Schwab-England Activities of Daily Living scale, Beck Depression Index (BDI), Health Survey Short Form (SF-36)	Improvements in SF-36 in cycling groups; Exergaming group had highest improvements in SE-ADL	Stationary + exergaming, Indoor
Tse et al. (143)	China	62	3 Groups (Learning to Ride a Bicycle Group: 5 sessions over 2 weeks, 60 min per session; Stationary Cycling Group: 2-wk stationary cycling program; Control Group: Activity as usual)	Cognitive	Tower of London (TOL), Corsi block tapping task (CBTT), Digit span, Stroop Test, Go/No-go (GNG)	Significant improvements in all executive function components and TOL Task in the learning to ride a bicycle group but not other groups	Bike + Stationary, Indoor
Vogt et al. (72)	Germany	11	2 Conditions (Cycle, Relaxation) for 10 min each; 2 sessions separated by 24 h.	Cognitive	EEG (N2), Reaction Time	Decreased frontal electrocortical activity, reaction time, and N2 latency after cycling.	Stationary, Indoor
Vogt et al. (96)	Germany	18	2 Groups (Moderate Intensity Cycling, No exercise); Three 5 min trials	Affective; Cognitive	EEG Cortical Activity, MoodMeter	Strong global alpha and beta responses when exercise was added	Stationary, Indoor
Webb et al. (57)	United States	16	2 Groups (Low-Fitness, High-Fitness); 2 Conditions (Exercise-alone condition: cycling at 60% V O2max for 37 min; Dual-challenge condition: mental challenge for 20 min while cycling); 3 Sessions (1 baseline, 2 experimental)	Affective; Cognitive	Stroop Test, Mental Arithmetic (MA) Task, State and Trait Anxiety Inventory (STAI); NASA Task Load Index (NTLX)	Dual Challenge Condition increased in state anxiety and exacerbated cortisol responses; LF participants had a greater overall cortisol response; Low Fitness Group had a greater cortisol response	Stationary, Indoor
Wu et al. (100)	China	16	Maximal exertion bicycle test; 2 sessions within 2 days in a low-load and a high-load condition	Cognitive	NIRS	Greater prefrontal activations in the low-load condition	Stationary, Indoor
Wunsch et al. (33)	Germany	84	2 Groups (Exercise: Cycle for 30 min moderate-vigorous intensity, Control: Stretch for 30 min)	Psychological; Social	Trier Social Stress Test for Groups (TSST-G), Salivary Stress Biomarkers (sCort and sAA)	Reduced stress activation for both types of exercise. Only habitual engagement in exercise exhibited a beneficial effect on peak cortisol levels	Stationary, Indoor
Yang et al. (144)	China	50	2 Groups (Aerobic Group: cycling training at 70% of maximal intensity for 40 min/d, 3 d/wk for 3 months, Control: health education)	Affective; Cognitive; Psychological	Mini-Mental State Examination (MMSE), APO-a1, Alzheimer's Disease Assessment Scale (ADAS-Cog), Quality of Life in Alzheimer's Disease (QoL-AD), Neuropsychiatric Inventory Questionnaire (NPI-Q)	MMSE score, QoL-AD, plasma Apo-a1 level significantly increased in cycling group; ADAS-Cog and NPI-Q significantly decreased	Stationary, Indoor
Zander et al. (32)	Australia	17	12-week cycling promotion program; cycle for at least 2 h	Affective; Psychological; Social	Qualitative Interviews	Interview themes: Choosing cycling for exercise; fear of cars and riding on streets; taking up cycling was a liberating, fun experience	Bike, Outdoor
Zink et al. (83)	Belgium	15	3 Conditions 12 min each (Free Pedal, Fixed Pedal, Still); 1 session	Cognitive	Oddball Auditory Task; EEG (P3 & N1)	Decrease in P300 amplitude in free biking condition	Stationary, Outdoor

The scope of inclusion was narrowed to studies in which bicycling (e.g., mountain biking, e-biking, road cycling, or stationary bicycling) was a primary intervention activity. Additionally, the study needed to assess bicycling's impact on a psychological, social, affective, and/or cognitive well-being outcome (see Table 1 for definitions of these domains).

In terms of exclusion criteria, gray literature and reviews (systematic, scoping, or narrative) were excluded to ensure all studies met the specific inclusion criteria of this review, although primary studies cited within relevant reviews were screened individually. Studies in which bicycling was incidental, secondary to another intervention, or where health outcomes could not be directly attributed to bicycling (i.e., combined effects of bicycling and walking) were excluded. Several records examining transportation and urban infrastructure were excluded for not meeting inclusion criteria, as they either did not include a specific intervention component, or did not assess one of the defined outcomes of interest. Several studies focused on competitive athletic performance were also excluded when bicycling was used to examine other outcomes, such as performance or heart rate, rather than the outcomes of interest. This review also excluded interventions that implemented stationary desk bikes and hand cycles.

### 2.3 Charting the data

Data extraction and charting were completed by Author LS with key characteristics of interest identified by the research team prior to data extraction. Data categories extracted included title, author, year of publication, country, sample size, participant age range, gender of population, whether race/ethnicity demographics were reported, whether a clinical population was studied, intervention time scale (acute vs. short-term vs. long-term), intervention details, selection of social, affective, psychological, or cognitive outcome measures, general results reported, type of bicycle, and whether the intervention occurred primarily indoors or outdoors. Acute interventions were defined as single or two-session interventions. Short-term interventions were defined as including at least three bicycling-specific sessions over at least two weeks and up to twelve weeks. Long-term interventions were defined as any intervention lasting more than twelve weeks. Each relevant outcome measure was coded as positive, negative, neutral, or inconclusive, with some studies including more than one outcome measure. Data extraction was verified by Author EW.

## 3 Results

### 3.1 Scientific literature search

**Figure 1:**
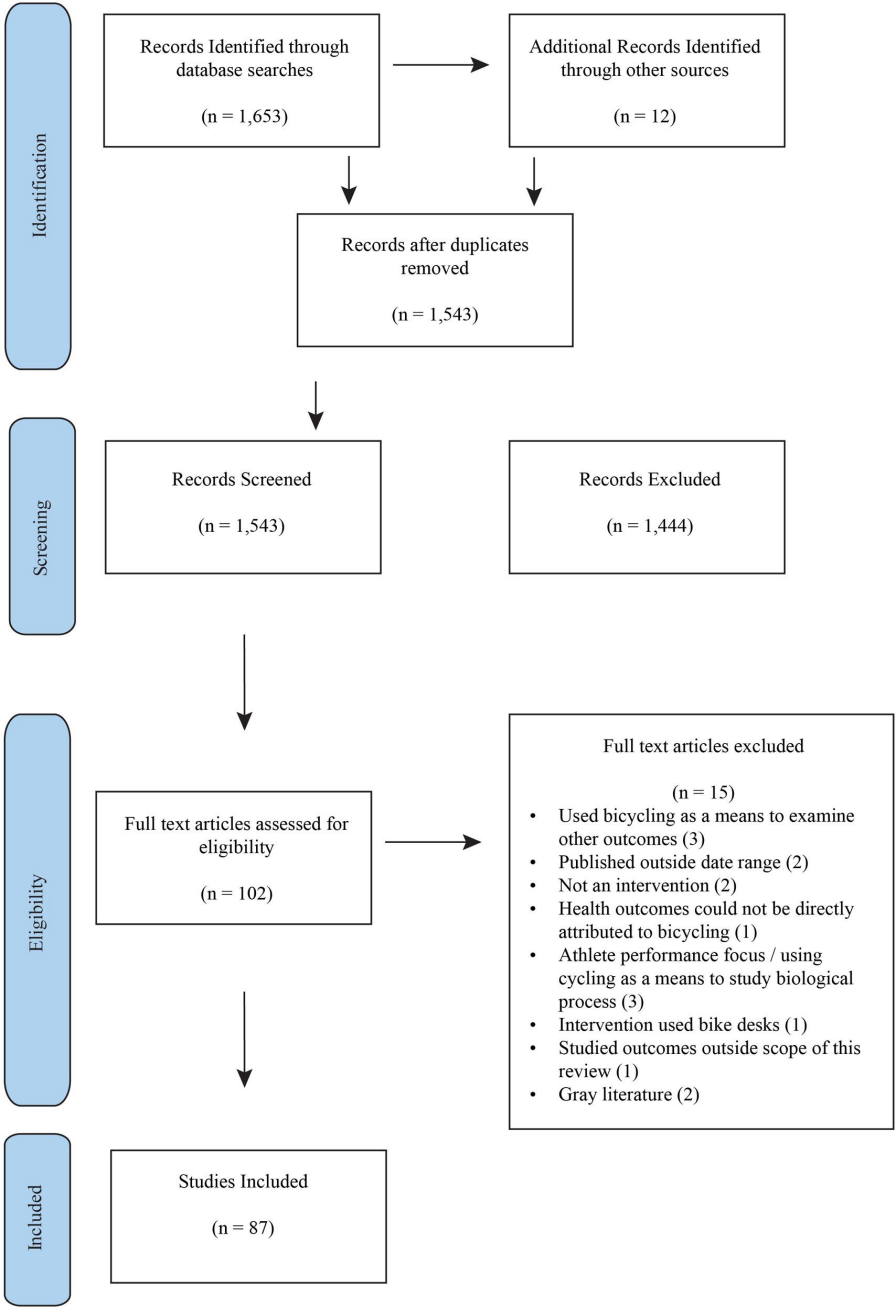
Study selection flowchart. Flowchart depicts the process of study selection through the stages of identification, screening, assessment of eligibility, and inclusion of studies in the final analysis.

A combined total of 1,653 studies were identified across all searches and imported into the Rayyan review management platform (18). After importing all studies, 110 duplicates were removed, leaving 1,543 studies to be screened. These studies underwent a blinded, independent screening of title and abstracts by two reviewers (Authors LS and BR). Conflicts were resolved through discussion with a third and fourth reviewer (Authors EW and CB) to reach consensus. Following screening, 102 full-text records were reviewed by the Author LS and assessed for inclusion eligibility. Of these, after full-text review, 15 records were excluded (See Figure 1). A total of 87 studies were included in the final scoping review for meeting the review's inclusion criteria and aim of investigating the effects of bicycling interventions on psychological, social, affective, and cognitive well-being (Figure 1).

### 3.2 Descriptive characteristics of included studies

Nineteen countries were represented among the 87 studies, with a total of 134 well-being outcomes represented across the studies (Table 2). The studies were conducted in the following countries: United States (*n* = 30; 35%), Germany (*n* = 16; 18%), Japan (*n* = 6; 7%), Australia and China (*n* = 5; 6%), Canada and the United Kingdom (*n* = 4; 5%), France (*n* = 3; 4%); Belgium, Italy, and South Africa each with 2 studies (2%) and Austria, Brazil, Colombia, Hungary, Netherlands, Republic of Korea, Russia, and Spain with 1 study (1%).

In terms of the population demographics, participant age ranges varied across studies, with 12% of studies including participants under 18 years of age, 83% of studies including adults between 18 and 55 years of age, 20% of studies including older adults greater than 55 years of age, and 15% of studies including two or more age groups. Among the included studies, 81% examined both males and females, 3% examined only females, 13% examined only males, 3% did not report on gender, and 2% included non-binary participants (Table 3). Only 5% of studies mentioned participant race and/or ethnicity.

**Table 3:** General characteristics of included studies. The table includes general characteristics of the 87 included studies across population age range, study design type, participant gender, and whether a clinical population was included. *Some studies included two or more age groups, causing percentages to total over 100%.

Category	Subcomponent	Number of Studies (%)
**Population Age Range**	Under 18 years old	11 (13%)
	Adults 18–55 years old	72 (83%)
	Older adults >55 years old	17 (20%)
	Two or more age groups	13 (15%) *
**Study Design**	Acute intervention	54 (62%)
	Short-term intervention	29 (33%)
	Long-term intervention	4 (5%)
**Gender**	Female and Male Participants	70 (81%)
	Female Participants only	3 (3%)
	Male Participants only	11 (13%)
	Did not report gender	3 (3%)
	Non-binary Participants	2 (2%)
**Population**	Not Clinical	61 (70%)
	Clinical Group	26 (30%)
**Race/Ethnicity**	Reported	4 (5%)
	Not Reported	83 (95%)

Clinical populations were the focus in 30% of studies including individuals with metabolic conditions (high BMI, pre-diabetes, obesity), neurological disorders (Parkinson’s disease, Cerebral Palsy, Alzheimer’s disease, Dementia, chronic post-stroke conditions), psychiatric conditions (Schizophrenia, methamphetamine use), intellectual or developmental disabilities (Down Syndrome, intellectual disability), cancer in elderly adults, persistent fatigue and diagnosed mobility limitations (Table 3).

In terms of study design, sample sizes within studies ranged from 7 to 1,148 participants. The majority of interventions were acute by design, with 62% coded as acute interventions, 33% coded as short-term interventions involving multiple sessions up to twelve weeks, and 5% of the included studies were long-term interventions with programming duration greater than 12 weeks (Table 3). Most studies utilized stationary bicycles for the intervention (84%), while fewer studies used 2-wheel bicycles (14%), e-bikes (5%), or a combination of bicycle types (2%). Additionally, 84% of studies were conducted indoors, while 16% of studies were conducted outdoors (Table 2).

### 3.3 Well-Being outcomes

#### 3.3.1 General psychological well-being

Psychological well-being outcomes were measured eighteen times, with 3 occurring in acute interventions, 13 in short-term interventions, and 2 in long-term interventions (Tables 2, 4). Of the studies, 67% reported positive impacts, 22% neutral, and 11% negative impacts of bicycling overall (Table 5). For acute studies, 33% reported positive results, 33% neutral, and 33% negative. Short-term interventions reported 77% positive results, 15% neutral, and 8% negative. For long-term interventions, one reported positive results and the other reported neutral results (Table 4). Indoor interventions reported 63% positive, 25% neutral, and 13% negative effects. Outdoor interventions reported similar results with 70% positive, 20% neutral, and 10% negative impacts (Table 6). Three studies (19–21) evaluated general psychological well-being using subscales from the 36-Item Short Form Health Survey (22) and found improvements among general well-being. Significant improvements in 36-Item Short Form Health Survey (SF-36) scores were found from pre to post intervention in the bicycling and high-intensity agility exergaming groups compared to the control group after 25 sessions (21). Neumeier et al. (20) reported significant positive changes in the intervention group, which actively commuted for 12 months, across four subcomponents of the SF-36 including physical functioning, mental health, vitality, and general health as well as the physical component summary score. Leyland et al. (19) reported a marginal interaction between session and group for the mental health component of the SF-36, with the e-bike cyclists increasing in this score, but no differences among pedal cyclists and non-bicycling controls. There was a significant improvement in mental health scores after the interventions, but this improvement did not differ between the groups.

**Table 4:** Summary outcome measures by study duration. Counts and proportions of outcome measures for each domain of well-being (psychological, social, affective, and cognitive), and whether the results were considered positive, neutral, negative, or inconclusive for study duration (acute, short-term, long-term). Note that each study may include more than one outcome measure and may include different outcome measures across different domains.

Well-Being Domain	Total	Acute					Short-Term					Long-Term				
		Positive	Neutral	Negative	Inconclusive	Total	Positive	Neutral	Negative	Inconclusive	Total	Positive	Neutral	Negative	Inconclusive	Total
	n	n (%)					n (%)					n (%)				
Psychological	18	1 (33%)	1 (33%)	1 (33%)	0 (0%)	3	10 (77%)	2 (15%)	1 (8%)	0 (0%)	13	1 (50%)	1 (50%)	0 (0%)	0 (0%)	2
Social	10	2 (100%)	0 (0%)	0 (0%)	0 (0%)	2	8 (100%)	0 (0%)	0 (0%)	0 (0%)	8	0 (0%)	0 (0%)	0 (0%)	0 (0%)	0
Affective	25	1 (10%)	3 (30%)	6 (60%)	0 (0%)	10	12 (86%)	2 (14%)	0 (0%)	0 (0%)	14	1 (100%)	0 (0%)	0 (0%)	0 (0%)	1
Cognitive	81	27 (45%)	12 (20%)	12 (20%)	9 (15%)	60	12 (67%)	3 (17%)	0 (0%)	3 (0%)	18	3 (100%)	0 (0%)	0 (0%)	0 (0%)	3

**Table 5:** Summary of results across well-being domains. Counts and proportions of outcome measures for each domain of well-being (psychological, social, affective, and cognitive), and whether the results were considered positive, neutral, negative, or inconclusive. Note that each study may include more than one outcome measure and may include different outcome measures across different domains.

Well-Being Domain	Positive	Neutral	Negative	Inconclusive
	n (%)			
Psychological	12 (67%)	4 (22%)	2 (11%)	0 (0%)
Social	10 (100%)	0 (0%)	0 (0%)	0 (0%)
Affective	14 (56%)	5 (20%)	6 (24%)	0 (0%)
Cognitive	42 (52%)	15 (19%)	12 (15%)	12 (15%)

**Table 6:** Summary outcome measures by domain and location. Counts and proportions of outcome measures for each domain of well-being (psychological, social, affective, and cognitive), and whether the results were positive, neutral, negative, or inconclusive by location in either the indoor or outdoor setting. Note that each study may include more than one outcome measure and may include different outcome measures across different domains.

Well-Being Domain	Total	Indoor					Outdoor				
		Positive	Neutral	Negative	Inconclusive	Total	Positive	Neutral	Negative	Inconclusive	Total
	n	n (%)					n (%)				
Psychological	18	5 (63%)	2 (25%)	1 (13%)	0 (0%)	8 (	7 (70%)	2 (20%)	1 (10%)	0 (0%)	10
Social	10	5 (100%)	0 (0%)	0 (0%)	0 (0%)	5	5 (100%)	0 (0%)	0 (0%)	0 (0%)	5
Affective	25	6 (35%)	5 (29%)	6 (35%)	0 (0%)	17	8 (100%)	0 (0%)	0 (0%)	0 (0%)	8
Cognitive	81	38 (50%)	14 (18%)	12 (16%)	12 (16%)	76	4 (80%)	1 (20%)	0 (0%)	0 (0%)	5

Four studies examined self-efficacy among participants using four different self-report measures. Two studies developed their measures following Bandura's recommended measurement approach (23). Samendinger et al. (24) demonstrated the importance of previous self-efficacy on future performance, while Focht et al. (25) found that sedentary adults and older adults reported lower self-efficacy after the bicycling intervention. One study (26) did not find significant effects of bicycling on self-efficacy when using a subscale of the Physical Activity and Self Efficacy Survey (27) while participants from Brookman et al. (28) had improvements in self-efficacy after a bicycling intervention when using the General Self Efficacy Scale (29). Combined, these results demonstrate that the set and setting may be important for self-efficacy and highlight the need for standardized use of self-efficacy questionnaires.

Ryu et al. (30) examined whether a 16-week bicycling intervention impacted well-being using the World Health Quality Assessment Instrument-100 (31) and reported no significant differences across an outdoor bicycling group when compared to an occupational therapy group of persons with Schizophrenia. Two studies using qualitative interviews also reported primary themes of improved well-being among participants (5, 32). Specifically, Brown et al. (5) reported themes of increased confidence and courage, motivation, and general mental well-being. Similarly, participants from Zander et al. (32) reported increased confidence after the intervention.

#### 3.3.2 Social well-being

Social well-being outcomes were measured 10 times, with 2 occurring in acute interventions and 8 in short-term interventions (Tables 2, 4). Two studies found positive impacts of acute interventions on social well-being, while 8 studies found positive impacts on social well-being in short-term interventions (Table 4). Of the 10 studies examining this well-being domain, 100% reported positive impacts of cycling on social well-being (Table 5), with 50% of interventions occurring indoors, and 50% occurring outdoors (Table 6). Six studies used a variety of self-report measures to capture different components of social well-being, including social isolation, sense of belonging, and community engagement. Five studies employed qualitative interviews where respondents reported on the impact of bicycling on social functioning.

There was a lack of consensus on how to measure social functioning, as each study employed a different instrument. Wunsch et al. (33) used the Trier Social Stress Test for Groups (34), Li et al. (35) used the Social Interaction Anxiety Scale (36) and The Prisoner's Dilemma Task (37), Geard et al. (38) used the VR-12 (39), Brookman et al. (28) used the Lubben Social Networking Scale (40), and Page & Nilsson (41) used The Flourishing Scale (42) and the GHQ-12 (43). Despite using different questionnaires, all studies reported that bicycling had positive effects on social well-being. Specifically, multi-session bicycling interventions may yield greater positive effects for expanding social network sizes and reducing loneliness (28, 38), while acute bicycling interventions may improve prosocial behaviors among participants (35).

Participants across five qualitative studies (5, 32, 44–46) described multiple social health benefits of bicycling including exposure to new friendships and community, improved social skills, increased feelings of connectedness, and a greater sense of “fitting in”. Overall, self-reported social well-being scores across the scales indicated that bicycling contributed to positive effects on social well-being.

#### 3.3.3 Affective well-being

Affective well-being outcomes were measured 25 times, with 10 occurring in acute interventions, 14 in short-term interventions, and 1 in a long-term intervention (Tables 2, 4). In total, 56% of studies found positive effects of bicycling on affective well-being, 20% neutral effects, and 24% negative effects (Table 5). Of the acute studies, 10% reported positive impacts on affective well-being and 30% neutral effects, with the majority of studies (60%) reporting negative impacts, though these interventions often induced exhaustion or fatigue (Table 4). For short-term interventions, 86% reported positive impacts and 14% neutral. One long-term intervention reported positive impacts. Of the indoor interventions, 35% reported positive results, 29% neutral, 35% negative. In contrast, 100% of outdoor interventions reported positive impacts on affective well-being (Table 6). Among the studies, 14 examined psychiatric symptoms, 5 assessed acute mood and affective states, and four employed qualitative interviews.

Eight studies examined the impact of bicycling on self-reported depression, with seven studies utilizing the Beck Depression Inventory (47) and one study utilizing the Geriatric Depression Scale (48). Three interventions reported improvements in depression symptoms scores, with lower scores indicating fewer symptoms. After a 16-week bicycling intervention, Ryu et al. (30) reported reductions in depression scores. Tollár et al. (21) reported reductions in BDI scores from pre to post intervention after a 5-week bicycling intervention that included thirty 1-hour sessions. Brookman et al. (28) found that post-intervention depression scores decreased or remained neutral for a majority of participants. Two studies did not find significant differences in depression scores, one employing an acute bicycling intervention (49) and the other a short-term intervention (50). Three studies (35, 51, 52) used the BDI as a baseline screening assessment.

Six studies examined participants’ self-reported anxiety during their bicycling intervention. Four of these implemented the State and Trait Anxiety Inventory (53), one used the Geriatric Anxiety Inventory (54), and one used the Beck Anxiety Inventory (55). One study measured anxiety solely at baseline (51). Three studies found no significant differences in anxiety from pre to post intervention (28, 50, 56). A 16-week program noted improvements for anxiety symptom scores (30). One study found that anxiety scores increased (i.e., symptoms worsened) in participants who completed a challenging mental task while they were bicycling (57).

Of the three studies employing variations of the Profile of Mood States (58), two found no differences in fatigue or energy states (56, 59). However, Dishman et al. (60) noted improvements in vigor following both low and moderate intensity exercise among participants with persistent fatigue.

Two studies examined affect through the Positive and Negative Affect Schedule (61). Leyland et al. (19) included the PANAS in their 8-week bicycling intervention for older adults and found a positive shift in PANAS scores across all groups (non-bicycling control, pedal bicyclists, and e-bike bicyclists). Szabo et al. (62) found acute positive changes in affect, with increases in positive PANAS scores and decreases in negative PANAS scores following a 35-minute indoor bicycling exercise.

Four studies utilizing qualitative interviews reported participant themes suggesting the bicycling program improved affective well-being. Main themes reported by Anderson et al. (44) included “feeling good,” “feeling happy,” and “more energy” following a 12-week e-bike loan intervention. Interviews reported by Zander et al. (32) revealed that female participants in particular, felt empowered and proud, and many reported improvements in energy and mood following the 12-week bicycling intervention. Parents in Armstrong et al. (45) reported their children with Cerebral Palsy demonstrated improved motivation, mood, and mindset following an 8-week training program. Similarly, qualitative interventions in Brown et al. (5) revealed themes of improved confidence, courage, and motivation among participants.

### 3.4 Cognitive well-being

Cognitive well-being outcomes were implemented 81 times, with 60 occurring in acute interventions, 18 in short-term interventions, and 3 in long-term interventions (Tables 2, 4). Forty-five percent of acute interventions reported positive impacts of bicycling on well-being, while 20% were neutral, 20% negative, and 15% inconclusive (Table 4). Of the short-term interventions, 67% reported positive impacts, 17% neutral, and 17% inconclusive. Three long-term interventions (100%) reported positive impacts of bicycling on cognitive well-being. Overall, 52% found positive impacts, 19% neutral, 15% negative, and 15% inconclusive impacts on cognition (Table 5).

With regards to location, of the indoor studies, 50% found positive impacts of bicycling on well-being, 18% neutral impacts, 16% negative impacts, and 16% were inconclusive. In contrast to the indoor interventions, 80% of outdoor interventions reported positive impacts of the bicycling intervention on cognition, and 20% of studies reported neutral impacts (Table 6). Thirty-seven studies used only behavioral measures of cognition, most frequently assessing processing speed (reaction time) and accuracy. Thirty-two studies used various methods to examine brain activity to understand bicycling's impact on cognitive function. Of these, twenty-five studies used electroencephalogram (EEG), five studies used functional near infrared spectroscopy (fNIRS), and two studies used functional magnetic resonance imaging (fMRI). Two studies combined behavioral responses with measures of brain-derived neurotrophic factor (BDNF). Below, we summarize key findings for both behavioral outcomes (reaction time, accuracy) and brain activity (EEG, fNIRS, fMRI).

#### 3.4.1 Behavioral outcomes

Researchers used a wide variety of tasks to assess cognitive outcomes before, during, or after bicycling. The most common tasks were the Stroop Task (11 studies), Auditory Oddball Task (9 studies), Wisconsin Card Sorting Task (7 studies), Flanker Task (6 studies), Trail Making Test (5 studies), Digit Span Test (5 studies), n-back Task (4 studies), and the Tower of London Task (3 studies).

##### 3.4.1.1 Reaction time

Nineteen studies examined processing speed by measuring reaction time on tests of executive function. Eleven acute interventions (63–73) noted improved reaction time after participating in bicycling interventions. Two acute interventions (51, 74) found no significant differences in reaction time. One acute intervention, designed to induce fatigue, noted impaired reaction time (75).

##### 3.4.1.2 Accuracy

Eight studies examined the impact of bicycling interventions on accuracy across various cognitive tasks. Three studies (19, 76, 77) reported significant improvements in accuracy after a bicycling intervention, while five studies (65, 66, 71, 74, 78) found no significant differences in accuracy. Even in an exercise-induced fatigue group that bicycled for one hour at a 90% ventilation threshold, accuracy did not differ from the rest group (75). Three studies (63, 69, 79) noted impaired accuracy. Additionally, Grego et al. (67) reported reduced accuracy after 120 min of bicycling.

#### 3.4.2 Brain activity

##### 3.4.2.1 Electroencephalogram and event related potentials

Electroencephalogram (EEG) is a technique used to capture electrical activity from the brain. EEG can also be used to generate event related potentials (ERPs) which measure changes in averaged EEG signals in response to specific stimuli or “events” and are often associated with both low-level sensory processing and cognitive processing (80). Fourteen studies measured ERPs, while 11 measured general cortical oscillatory activity (Table 2). Twenty studies examined cortical activity *during* bicycling, 5 compared activity before and after bicycling, and 5 compared activity during and before or after bicycling. Out of the 25 studies, 22 were conducted in an indoor setting using stationary bicycles. Findings regarding the impact *while* bicycling on ERP components have also been reported in a previous Systematic Review (81).

Eleven studies examined the P300 ERP component (51, 65, 69, 76, 78, 79, 82–84, 86, 125). The P300 component is typically elicited using oddball paradigms, in which responses to less common stimuli in the midst of repetitive stimuli are recorded and is commonly interpreted as reflecting attentional allocation and context updating (85). Larger amplitudes generally reflect greater allocation of attentional resources, whereas longer latencies may indicate slower stimulus evaluation. Environmental complexity (e.g., traffic) and cadence modulated P300 effects, with high-load environments reducing attentional allocation and reducing P300 amplitude (78) and optimal cadence enhancing attention compared to fast and slow conditions (82). In total, seven studies found increases in P300 amplitudes during or after bicycling (51, 65, 67, 69, 76, 79, 84), and four found decreases in P300 amplitude (78, 82, 83, 86).

Four studies examined the N1 component (78, 79, 83, 86). The N1 component reflects early sensory and attentional processing and is sensitive to stimulus discrimination and selective attention (87). In total, two studies found increases in N1 amplitude, one found no differences, and one study found a decrease. N1 amplitude was larger in high-traffic environments (78, 86), consistent with enhanced early sensory processing under conditions of increased environmental vigilance. Conversely, one study assessing ERPs after a bout of bicycling found reduced N1 amplitud**e** at post-test relative to rest (79), which may reflect temporary post-exercise fatigue. Importantly, these differences highlight that during-exercise and post-exercise effects may not be directly comparable.

Four studies examined the N2 component (69, 72, 79, 88). The N2 component is often associated with conflict monitoring, response inhibition, and aspects of cognitive control (89). Overall, N2 amplitude decreased during low or vigorous cycling in three studies. Moderate cycling neutralized the N2 in one study. Two studies reported shorter N2 latencies following bicycling (72, 79), a pattern sometimes interpreted as more efficient conflict monitoring. Another study found greater negative N2 amplitudes after two bicycling conditions compared to rest (69), suggesting stronger cognitive control. Dodwell et al. (88), examined the posterior-contralateral negativity (PCN), a sub-type of the N2, and found an inverted-U relationship where rest and vigorous bicycling reduced its amplitude or increased the latency, while moderate bicycling eliminated these effects.

One study examined the N400 component (90), a marker of **s**emantic processing, and found an increase in amplitude (91). During a vocabulary-encoding task performed while bicycling, N400 amplitude increased relative to a non-bicycling condition (90). This suggests that concurrent physical activity can modulate the neural resources recruited for semantic encoding, although more work is needed to determine whether bicycling enhances or increases the demands of semantic processing.

In addition to ERPs, 11 studies examined cortical oscillatory activity through EEG (Table 2). Across these studies, alpha power commonly increased during warm-up and moderate bicycling (7 studies), which may reflect shifts toward more internally oriented attention or reduced visual processing demands (60, 65, 92–96). At higher exercise intensities, two studies reported decreases in alpha power (93, 97), a pattern typically interpreted as greater cortical activation and heightened task engagement. One study similarly reported reduced alpha activity during higher intensity bicycling compared to lower intensity bicycling (60). In addition to alpha activity, increases in beta and gamma power were observed during bicycling, particularly in frontal and parietal regions, and were associated with enhanced motor and attentional control (92).

##### 3.4.2.2 Functional near-infrared spectroscopy

Five studies used functional near-infrared spectroscopy (fNIRS) to examine brain activity during bicycling (Table 2). One study demonstrated that fNIRS is a feasible and valid method for measuring cortical activation *during bi*cycling, showing that hemodynamic responses associated with both motor and cognitive function changed with exercise intensity (98). Two other studies found changes in brain activation across brain regions including the prefrontal cortex and primary motor cortex (99, 100), consistent with executive functioning and sustained attentional engagement.

##### 3.4.2.3 Functional magnetic resonance imaging

Two studies used functional magnetic resonance imaging (fMRI) to examine neural activity in relation to bicycling. One study reported changes in activation in the cerebellum, precentral gyrus, and postcentral gyrus during periods of bicycling (101). The other study found that brain activity during successful spatial learning was modulated by the participant's physical fitness across a 6-month intervention comparing a bicycling (aerobic activity) group to a stretching group (102).

## 4 Discussion

### 4.1 Summary

This scoping review includes synthesized evidence from 87 intervention studies performed across 19 countries, emphasizing the multidimensional potential of bicycling interventions to promote well-being across psychological, social, affective, and cognitive dimensions. Summarized results across all studies demonstrated that bicycling interventions have a majority of positive effects on the different domains of well-being across both indoor and outdoor environments, as well as across different lengths of interventions. Positive results were more consistent among outdoor and short- and long-term bicycling interventions. While several studies reported negative effects of bicycling in the indoor environments, those study protocols often included tests to exhaustion, or vigorous cycling, demonstrating an inverted- U effect of bicycling in the acute high intensity settings indoors. These study protocols were intentionally designed to induce negative valence and to challenge participants, leading to the higher proportions of negative impacts reported among acute interventions. Inconclusive results were reported for studies that only included a brain imaging measure, where it could not be determined if the effect of bicycling was positive, neutral, or negative.

Similarly, in a scoping review focused on mountain biking, Kuklinski et al. (103) identified 116 studies spanning 24 countries that were used to identify five domains of influence (i.e., physical, psychological, social, environmental, and cognitive). Their analysis highlighted how mountain biking research increasingly conceptualizes bicycling as a complex biopsychosocial phenomenon rather than a purely recreational or physical pursuit. With the expansion of our review to focus more broadly on all bicycling-based interventions, the convergence between the reviews further supports bicycling's relevance for emotional regulation, stress reduction, and social connectedness. Several patterns emerge from our review, illuminating the holistic impact of bicycling on human well-being, including improvements in participant mental health, depressive symptomology, mood, and cognitive processes. Taken together, this impact may be attributed to factors above and beyond physical activity, including several unique features of bicycling such as outdoor exposure, opportunities for social interaction, and engagement in meaningful routines (1, 2).

#### 4.1.1 Psychological

Across the reviewed studies, bicycling's impact on psychological well-being, particularly self-efficacy, was context dependent. One intervention demonstrated improved self-efficacy using the validated General Self-Efficacy Scale (29) after a bicycling intervention, consistent with findings from Brown et al. (5, 104), who reported enhanced self-efficacy among middle school students in mountain bike programs. However, other studies showed a mix of positive, neutral, and even contra-therapeutic outcomes, particularly among sedentary adults and older populations (25). This variability underscores the critical importance of “set and setting” in intervention design, suggesting that outcomes are significantly influenced by the psychological and environmental context in which bicycling occurs. It also highlights the importance of designing interventions for older adults that reduce barriers to physical exercise and that make it appealing, particularly for those who have not recently engaged in physical activity. Together, these findings suggest that bicycling interventions are most effective when they foster self-efficacy and align with self-determination theory, supporting autonomy, competence, and relatedness, key drivers of sustained motivation and well-being.

While general mental health outcomes were not frequently reported in this review, three studies that did measure general mental health reported positive increases (19–21). Notably, these studies were not solely acute in nature, with programming spanning the course of several weeks. The results indicate that general mental health may be more positively impacted by interventions with multi-session programming compared to acute programming, which is consistent with previous work demonstrating a dose-response relationship between physical activity and mental health (105). Future work should examine optimal program length to achieve benefits, as this can be important for practitioners to incorporate into program design.

Together, these findings align with preexisting literature emphasizing psychological well-being as a multidimensional construct encompassing self-acceptance, environmental mastery, autonomy, and personal growth (106, 107). This supports the assertion that interventions focused on fostering psychological well-being can lead to better coping strategies and improved mental health outcomes which can be leveraged to inform future program development and research (1). Previous work suggests techniques for practitioners to adjust programming to enhance self-efficacy by setting challenging but reachable goals that build on each other, creating social groups within an intervention to encourage participation, incorporating self-regulation techniques, and reducing exacerbation of physical limitations (108). The adaptability of bicycling factors such as riding location, bicycle type, and opportunities for social engagement support its effectiveness as a tool from which self-efficacy interventions can be tailored. Indeed, Kuklinski et al. (103) highlighted self-efficacy and perceived competence as the most frequently cited psychological constructs across mountain biking studies, with outcomes strongly influenced by environmental conditions, risk perception, and social support. They found that experiences fostering mastery and autonomy, particularly in nature-rich settings, were associated with improved confidence, vitality, and identity development.

#### 4.1.2 Social

Although social outcomes were only explored among 10 measures, the findings are encouraging. Participants in short-term bicycling interventions consistently demonstrated the ability to expand social networks and reduce loneliness, while those in acute interventions showed potential to foster prosocial behaviors. Additionally, benefits to social well-being were found across both indoor and outdoor studies, highlighting how stationary bikes can also facilitate opportunities for social engagement. Qualitative findings suggested powerful themes around community integration, skill development, and social connectedness, consistent with prior research demonstrating that social interactions and engagement in community activities facilitated through bicycling contribute to greater social cohesion and a sense of belonging (5, 9, 104). These findings support the conceptualization of social well-being as encompassing the quality of an individual's relationships and interactions within their community, including intrapersonal feelings of belonging and connection (103, 109). Kuklinski et al. (103) found that group-based riding promotes prosocial identity formation and inclusion, particularly in participants in youth programs.

#### 4.1.3 Affective

In terms of affective well-being, findings indicated differential effects between depression and anxiety outcomes. Depressive symptoms demonstrated consistent improvement across multiple studies using validated instruments, supporting prior research linking physical activity, including bicycling, to reduced depressive symptoms and enhanced mood (6). Neurotransmitter secretion during physical activity, such as bicycling, has been linked to improved emotional well-being (7), potentially explaining the consistent improvements in depressive symptoms observed across studies.

Observed changes in anxiety symptoms after bicycling, on the other hand, were more variable and appeared to be context dependent. For instance, dual-task paradigms, such as bicycling while performing a cognitive task, have been shown to increase anxiety levels, suggesting that cognitive demands during bicycling may moderate affective outcomes (57). Many of the acute studies incorporated anxiety or stress inducing components within their interventions, contributing to the higher proportion of negative results among affective well-being. Kuklinski et al. (103) reported similar trends in their review, showing that moderate challenge and outdoor exposure during mountain biking enhance affective states through flow state and self-determination, whereas excessive demands, such as a poorly designed or overly competitive environments, could potentially elicit anxiety or fear, further illustrating the importance of emotional regulation and perceived control in bicycling-based interventions. Multi-session interventions were distinct from acute sessions as they reported higher proportions of positive effects of bicycling on improving mood and psychiatric symptoms. These interventions often did not include components that induced negative emotional states within participants, leading to higher proportions of positive results. Similar contrasts were found between outdoor and indoor interventions. These findings highlight the complexity of affective well-being, encompassing both the presence of positive emotions, such as joy and contentment, and the absence of negative emotions, such as anxiety and despair (110).

#### 4.1.4 Cognitive

In relation to domains of cognitive functioning, several patterns emerged that illustrate the holistic impact of bicycling on well-being. Processing speed on a variety of cognitive tasks improved after participating in both acute and short-term bicycling interventions, though accuracy largely remained unchanged. This aligns with research suggesting the positive impact of physical activity on cognitive processes and executive function (111).

Across event related potential (ERPs) components, high environmental load (e.g., busy traffic, complex surroundings) appears to enhance early sensory processing (increased N1) while reducing later attentional allocation (reduced P300). In contrast, moderate-intensity or optimal-cadence bicycling may support or enhance cognitive control processes, whereas high-intensity bicycling or fatigue-inducing protocols tend to impair attentional allocation in acute settings. This aligns with an “inverted-U-shaped” relationship between bicycling intensity and cognitive performance, where moderate levels of stimulation optimized neural efficiency while excessive or insufficient intensity may have impaired cognitive outcomes (88). This phenomenon was also observed in behavioral measures (75). Similar to affective well-being results, multi-session studies reported higher proportions of positive outcomes on cognitive well-being, highlighting how consistent bicycling over time can support brain health and cognitive function. Altogether, these findings suggest that the impact of bicycling on neural processing is influenced by environmental complexity, exercise intensity, consistency, and task demands.

Cognitive outcomes were overrepresented in this scoping review, appearing in most studies. However, many of these studies were conducted indoors on stationary bicycles, highlighting the need for translational research to better understand how cognition may be impacted from bicycling outdoors. Indeed, Kuklinski et al. (103) noted that only 4% of included outdoor mountain biking studies employed objective neurocognitive measures, highlighting a critical gap for future research. Promising results from Tempest and Reiss (98) validated fNIRS as a solution to understanding brain activity while bicycling outside of the lab and found that bicycling modulates cognitive processing. Additionally, 15 studies using EEG were conducted *during* bicycling, providing evidence that measuring meaningful brain activity during exercise is possible.

These findings underscore the importance of conceptualizing cognitive processing as distinct from, yet connected to, psychological and affective responses, as effective cognitive functioning facilitates emotional regulation and supports overall mental health (110). Furthermore, research supports the notion that bicycling interventions that seek to enhance cognitive processing can significantly contribute to both learning outcomes and psychological development among participants across various contexts (112).

#### 4.1.5 Additional considerations

As noted in the results, almost all included studies did not report on participants’ race or ethnicity. Reporting participant racial and ethnic demographics is critical for understanding how bicycling interventions may differently impact people across diverse backgrounds. Previous research has demonstrated lower rates of bicycling participation amongst racial and ethnic minorities in addition to older adults who own bicycles (113). Individuals who do not currently identify as bicyclists may experience greater health benefits if they adopt bicycling as a regular lifestyle behavior. Similarly, promising results indicate that bicycling programs can benefit women, who typically participate at lower rates than men, especially in areas where cycling mode share is low (114), by improving mood, confidence, and related outcomes (32). Future studies should include comparative analyses across gender and explore tailored strategies to address barriers that may differ across groups.

Adolescents and older adults were underrepresented in the studies reviewed, yet both groups face critical life transitions that affect well-being. Cycling programs may offer meaningful benefits, including improved focus and well-being in youth (5) and enhanced cognitive function, mobility, and mental health in older adults (115). Including these populations is essential to understand cycling's benefits across the lifespan, and program design should account for age-specific considerations and barriers, such as safety, motivators, and access to appropriate equipment. Improving access to bicycling and engaging underrepresented populations in research is crucial for understanding how bicycling impacts well-being domains across different groups, while also identifying the associated barriers and facilitators.

Community bicycling initiatives and bicycling-related non-profits can play an essential role in expanding access to bicycling and its associated benefits. Clanton et al. (15) analyzed how local organizations advance community well-being through bicycling and found that they not only support bicycling itself but also can enhance community well-being across multiple domains. This emphasizes the importance of community-based participatory research, connecting researchers and bicycling organizations, to better understand how real-world bicycling programs impact the well-being of individuals and their communities. Currently published program evaluations and studies of these initiatives demonstrate promising results (15, 116), however, there is a need for additional research on bicycling interventions, examining all domains of well-being and how they interact. Despite the majority of included studies examining cognitive outcomes, many of these were acute, on stationary bikes, and in a lab setting. There is opportunity to translate this research into real-world interventions, especially on the impacts of bicycling interventions on the cognition of older adults, as recent work has highlighted how improving lifestyle factors, like increased physical activity, can improve cognition (117).

### 4.2 Limitations and future directions

One methodological limitation of this review arose during our search for relevant studies. The term “cycling” was removed from the key terms list because it connotes biological cycle processes. In addition, specific types of bicycles (e.g., e-bikes, tricycles) were not included among our search terms. However, several articles including these types of bikes underwent the screening process. To refine our search, we added in the MeSH terms when available to improve study identification. In addition, bicycling is often referred to as active travel, aerobic exercise, or physical exercise. To improve future searches, authors should ensure appropriate indexing and clearly define the type of physical activity implemented (i.e., bicycling, stationary bike) to improve discoverability and accessibility of the literature.

While most studies employed acute interventions using stationary bicycles within a lab setting with adult age groups, future research has exciting opportunities to expand understanding of how bicycling interventions impact domains of well-being. Moving beyond lab settings allows researchers opportunities to examine the potential long-term benefits in ecological contexts, such as outdoor bicycling and active transportation, capturing sustained, real-world benefits beyond acute exercise.

Finally, a shift toward naturalistic settings and diverse population sampling, including youth, older adults, racial and ethnic minorities, and individuals with differing abilities or clinical conditions, will strengthen knowledge of bicycling's therapeutic potential across the lifespan. Results from studies engaging clinical populations, such as participants with neurological disorders or metabolic disease, highlight bicycling as a promising therapeutic intervention, however, additional research should replicate and build upon current findings. While many of the included studies assessed the cognitive domain of well-being, fewer studies examined psychological, social, and affective outcomes on well-being. Incorporating objective measures alongside self-report instruments targeting these less studied domains would enhance comparability across studies and reduce potential bias, ultimately building a more robust evidence base for bicycling interventions. Considering the post-pandemic shifts in bicycling patterns and community perceptions (118, 119), longitudinal research examining sustained engagement and long-term outcomes would be particularly valuable.

### 4.3 Conclusions

Based on this scoping review, bicycling interventions demonstrate significant promise for promoting well-being across psychological, affective, social, and cognitive domains. While a majority of studies emphasized cognitive effects within an indoor environment, a growing number of studies explored psychological, social, and affective outcomes. Overall, though fewer studies were conducted in outdoor settings, a high percentage of studies reported positive effects regardless of setting. Evidence suggests that bicycling's benefits extend beyond physical health (2, 3, 5, 6) to encompass emotional regulation, stress reduction, social connectedness, and cognitive enhancement. However, realizing this potential requires intentional program design, equitable infrastructure development, and continued research across populations in real-world settings. By strategically implementing bicycling interventions within educational, clinical, and community contexts, we can leverage this accessible, low-cost activity to address pressing public health challenges while fostering individual and community well-being. As bicycling continues to gain recognition as both a practical mode of transportation and a therapeutic intervention, stakeholders across sectors have an opportunity to harness its holistic benefits to promote health equity and enhance quality of life across diverse populations.

## Data Availability

The original contributions presented in the study are included in the article/Supplementary Material, further inquiries can be directed to the corresponding author.
